# Placental Transfer and Composition of Perfluoroalkyl Substances (PFASs): A Korean Birth Panel of Parent-Infant Triads

**DOI:** 10.3390/toxics9070168

**Published:** 2021-07-14

**Authors:** Habyeong Kang, Hee-Sun Kim, Yeong Sook Yoon, Jeongsun Lee, Younglim Kho, Jisun Lee, Hye Jin Chang, Yoon Hee Cho, Young Ah Kim

**Affiliations:** 1 Department of Environmental Health Sciences, School of Public Health, Seoul National University, Seoul 08826, Korea; habyeonk@umich.edu; 2 Department of Epidemiology, School of Public Health, University of Michigan, Ann Arbor, MI 48109, USA; 3 Departments of Obstetrics and Gynecology, Dongguk University Ilsan Hospital, Goyang-si 10326, Korea; smallkong7@gmail.com; 4 Center for Health Promotion, Departments of Family Medicine, Inje University Ilsan Paik Hospital, Goyang-si 10380, Korea; ysyoon@paik.ac.kr; 5 Department of Health, Environment and Safety, School of Human & Environmental Service, Eulji University, Seongnam-si 13135, Korea; dlwjdtjs2121@naver.com (J.L.); ylkho@eulji.ac.kr (Y.K.); 6 Department of Obstetrics and Gynecology, Kyungpook National University Hospital, School of Medicine, Kyungpook National University, Daegu 41944, Korea; vmariagnes@gmail.com; 7 Department of Obstetrics and Gynecology, Ajou University Hospital, Ajou University School of Medicine, Suwon-si 16499, Korea; zzanga-94@ajou.ac.kr; 8 Department of Biomedical and Pharmaceutical Sciences, The University of Montana, Missoula, MT 59812, USA; 9 Departments of Obstetrics and Gynecology, Inje University, Ilsan Paik Hospital, Goyang-si 10380, Korea

**Keywords:** perfluoroalkyl acids, cord blood, placenta, pregnancy, prenatal exposure

## Abstract

Exposure to perfluoroalkyl substances (PFASs) is of public concern due to their persistent exposure and adverse health effects. Placental transfer of PFASs is an important excretion pathway of these chemicals in pregnant women and exposure route in fetuses. We measured PFAS concentrations in maternal, paternal, and umbilical cord serum collected from 62 pregnant Korean women and matched biological fathers of the fetuses. Placental transfer rates (cord to maternal serum ratio) of PFASs were also calculated. Demographics and pregnancy-related factors determining the placental transfer rates were identified using linear regression models. Maternal, paternal, and cord serum showed different PFASs compositions. Among the PFASs, perfluorooctane sulfonate (PFOS) showed the highest concentrations in maternal and paternal serum, while perfluorooctanoic acid (PFOA) showed the highest concentration in cord serum. There was a higher proportion of perfluoroalkyl carboxylic acids (PFCAs) with 9–12 carbon chains than those with 13–14 carbon chains in maternal and paternal serum, but this proportion was in the opposite direction in cord serum. PFOA and perfluorohexane sulfonate (PFHxS) had higher placental transfer rates (means of 0.32 and 0.36, respectively) than PFOS (mean of 0.12), which is in line with the results of previous studies. Gestational age and birth weight were positively associated with placental transfer rate of PFOA, PFHxS, and PFOS, while pre-pregnant BMI and weight were inversely associated with PFOS. This study showed that placental transfer of PFASs differs by compounds and is associated with pregnancy-related factors. Further studies on novel PFASs are warranted for Korean pregnant women.

## 1. Introduction

Perfluoroalkyl substances (PFASs) are a group of compounds with a fully fluorinated carbon chain. PFASs have both hydrophilic and hydrophobic properties; thus, they are used in various products including food packing, cookware, fire-fighting foams, alkaline cleaners, floor polishes, photographic films, shampoos, insecticides, soil- and stain-resistant coatings for fabrics, and grease- and oil-resistant coatings for paper products [1,2]. Due to the wide range of PFASs usage, people have been ubiquitously exposed to PFASs from their living environment [3], consumer products [4,5], drinking water [6], and foods [7].

Toxicological and epidemiologic studies have found various adverse health effects of PFASs [8]. Several PFASs have long biological half-lives, which make them more harmful to humans. For example, perfluorohexane sulfonate (PFHxS) and perfluorooctane sulfonate (PFOS) have been estimated to have 5.3–8.5 years and 3.4–5.0 years of half-lives, respectively, and half-lives of perfluorooctanoic acid (PFHpA), perfluorooctanoic acid (PFOA), and perfluorononanoic acid (PFNA) have been estimated to range 1.2–4.3 years [8].

There have been efforts to reduce or phase out PFAS in the industries in order to minimize PFASs-induced health risks. From 2002, 3M, the primary global manufacturer of PFOS, voluntarily tried to cease its production containing PFOS [9]. Further efforts to reduce global PFOS emissions by placing restrictions on its use and marketing were reinforced in 2006 by the European Union (EU) [10] and in 2009 by the Stockholm Convention [11]. In 2006, under the United States Environmental Protection Agency’s (US EPA) 2006 PFOA Stewardship Program, eight of the largest users and producers of PFOA, which included Arkema, 3M, Asahi, Dupont, BASF Corporation, Clariant, Daikin, and Solvay Solexis, committed to reduce global emissions and use of PFOA, its precursors, and other long-chain PFAS by 95% by 2010 and eliminate them by 2015 [12]. Some companies also participated and committed to reduce these substances by 2013 [13]. These efforts have gradually reduced the body burden of PFOS and PFOA in the general population [14,15,16]. However, exposure to PFASs with longer chains such as perfluoroundecanoic acid (PFUnDA) and perfluorododecanoic acid (PFDoDA) has increased [16], and major PFASs such as PFOA, PFNA, PFHxS, and PFOS are still found in most human sera in detectable levels [4,17] due to their long biological half-lives and persistent exposure.

Several environmental pollutants (known as endocrine-disrupting chemicals), including pesticides, polychlorinated biphenyl (PCBs), phenols, phthalates, and dioxin are reported to affect folliculogenesis [18,19,20,21,22]; however, recently published literature has shown insufficient associations between PFAS exposure and ovarian disorders [18]. In addition, prenatal exposure to PFASs can be a critical window since early-life PFAS exposure can cause pregnancy-induced hypertension, preeclampsia, gestational diabetes, and low birth weight, which is caused by placental insufficiency [23]. PFASs have been known to be transferred to fetuses via the placenta. This placental transfer has been suggested as an excretion route of PFASs in pregnant women as well as an exposure route in fetuses [24]. Previous studies have reported that placental transfer varies by PFAS compound [25], and the carbon number of the PFASs was suggested as a factor to explain the difference in placental transfer rate [26]. Thus, placenta is one of the target tissues after PFAS exposure and plays a role in the etiology of pregnancy disorders. However, the placental transfer rate of PFASs and their determinants are not fully understood yet.

Furthermore, several studies have reported the effects of paternal exposure to persistent organic pollutants (POPs) on birth outcomes of offspring. A study by Robledo et al. reported the effect of paternal exposure to PCBs on lower birth weight of offspring [27]. Several studies also showed that long-chain perfluoroalkyl carboxylic acids (PFCA) exposure affected semen quality and spermatogenesis [28,29] in men, and paternal exposure to PFASs, including PFNA and *N*-methylperfluorooctane sulfonamidoacetic acid (MeFOSAA), has been associated with different secondary sex ratio (i.e., an excess of female birth [30]). These results suggest that paternal exposure to POPs, including PFCAs, may contribute to health outcomes in offspring just as maternal exposure does. However, evidence-based studies on paternal exposure to PFASs and its potential to affect birth outcomes in offspring have not been extensively established yet.

Therefore, in the present study, compositions of the PFASs in maternal, paternal, and cord serum were compared using serum samples collected from Korean pregnant women and their husbands. We also investigated the placental transfer of PFASs and their determinant factors using PFASs concentrations in maternal and cord serum.

## 2. Materials and Methods

### 2.1. Study Population and Sample Collection

From March 2013 to July 2015, 65 pregnant women at 24–42 weeks of gestation and their husbands were recruited in Inje University Ilsan Paik Hospital in South Korea. When the participating couples visited the hospital for their delivery, data on demographic characteristics (e.g., age, family income, and education level) and health status (e.g., smoking history, weight, and parity) of the participants were collected by questionnaire survey. Birth outcomes were obtained from medical records. When the questionnaire survey was conducted, blood was also collected from the pregnant women and their male partners (biological fathers). Umbilical cord blood was collected during the delivery. Serum and buffy coat were separated from the blood samples, and they were stored at −80 °C until analyses. Among the 65 couples, 3 couples without both maternal and cord blood measurement were excluded in the present study. Furthermore, 10 paternal serum samples were also insufficient to analyze PFAS; thus, 62 paired mothers and newborns as well as 52 biological fathers were finally included for this study. During the questionnaire survey, written consent was obtained from the participants. A protocol of this study was reviewed and approved by the Institutional Review Board of Inje University Ilsan Paik Hospital (2012-11-138).

### 2.2. Analysis of PFASs in Serum and Quality Assurance

In the serum samples, 13 perfluoroalkyl acids, which have been targeted in many previous biomonitoring studies [31,32,33,34], were analyzed following a method in previous studies [35,36]. The target PFASs included PFCAs (i.e., perfluoropentanoic acid (PFPeA), perfluorohexanoic acid (PFHxA), PFHpA, PFOA, PFNA, perfluorodecanoic acid (PFDA), PFUnDA, PFDoDA, perfluorotridecanoic acid (PFTrDA), and perfluorotetradecanoic acid (PFTeDA)) and perfluoroalkane sulfonates (PFSAs) (i.e., perfluorobutane sulfonate (PFBS), PFHxS, and PFOS). In 200 μL of a serum sample, 20 μL of labeled internal standards were spiked. PFASs were extracted from Oasis mixed-mode weak anion-exchange solid-phase extraction cartridges (1 cc/30 mg, Waters, Milford, MA). The analytes were eluted with 4 mL of 0.1% ammonium hydroxide in methanol. Then, the extract was dried and reconstituted in 200 μL of acetonitrile.

Identification and quantification of PFASs were processed by high-performance liquid chromatography (HPLC series 1100 system, Agilent Technologies, Palo Alto, CA) coupled with tandem mass spectrometry (API 4000, Applied Biosystems, Foster City, CA). Separation of PFASs was performed using a C18 column (YMC-Pack ODS-AQ, 2.0 × 150 mm, 3.0 μm, Waters, Milford, MA). Then, 3 μL of each sample was injected. The flow rate was 200 μL/min. Electrospray ionization negative mode with multiple reaction monitoring was used for the detection of PFASs. Details of the analytical conditions and limits of detection (LODs) of PFASs are shown in Supplemental Tables S1–S3.

### 2.3. Statistical Analysis

Non-detects were replaced with a proxy value of LOD divided by √2 when PFASs were detected in >80% of the samples. Otherwise, they were replaced with zero [37]. Descriptive statistics, i.e., means (geometric means for PFASs concentrations and arithmetic means for other variables), standard deviation, and percentiles, were calculated. The sum of PFCA concentrations (∑PFCAs) was calculated as a sum of PFPeA, PFHxA, PFHpA, PFOA, PFNA, PFDA, PFUnDA, PFDoDA, PFTrDA, and PFTeDA concentrations. The sum of PFSAs concentrations (∑PFSAs) was calculated as a sum of PFBS, PFHxS, and PFOS concentrations. The sum of PFASs concentrations (∑PFASs) was calculated as a sum of ∑PFCAs and ∑PFSAs. Correlation among the PFASs in maternal, paternal, and cord serum was determined by Spearman’s correlation analysis. Based on the arithmetic mean concentration of each PFAS, their compositions in the maternal, paternal, and cord serum were calculated. The placental transfer rate was calculated by dividing the concentration in cord serum by the concentration in maternal serum. Linear regression was conducted to identify the determinants of cord and maternal serum concentrations of PFASs or their placental transfer rates. Considering the right-skewed distribution, concentrations of PFASs were log-transformed for the linear regression. For continuous variables (i.e., maternal age, pre-pregnant body-mass index (BMI), pre-pregnant weight, weight gain during the pregnancy, birth weight, and gestational age), the effect size was estimated for an interquartile range (IQR) increase in each independent variable and represented as a difference in a placental transfer rate of each PFAS [38]. Infant sex and parity were categorized to estimate their effect on placental transfer rates. A *p*-value < 0.05 was considered to be statistically significant. SAS (version 9.4, SAS Institute, Cary, NC) and R software (version 3.6.1, R Foundation for Statistical Computing, Vienna, Austria) were used for the statistical analysis.

## 3. Results

### 3.1. Demographic Characteristics of the Study Population

A total of 62 couples were included in this study with 10 male partners excluded because of missing and/or insufficient values, which resulted in a total of 52 fathers in the analysis (Table 1). The arithmetic mean ages of female and male partners were 32 and 34 years, respectively. About 80% of both female and male participants had > 12 years of education. Additionally, 55% of the women were nulliparous at the recruitment. There were no current smokers among the female partners, and their pre-pregnant BMI ranged from 16.6 to 32.2 kg/m^2^. The average birth weight of their infants was 2.94 kg with a range of 1.71–3.76 kg. Their gestational age ranged from 30.6 to 41.0 weeks, and 56% of the infants were male.

### 3.2. Detection of PFASs in Maternal, Paternal, and Cord Serum

Among the target PFASs, PFOS showed the highest concentration in maternal and paternal serum (geometric means of 2.31 and 2.21 ng/mL, respectively) while PFOA showed the highest concentration in cord serum (geometric mean of 0.40 ng/mL; Table 2). PFOA, PFHxS, and PFOS were detected in most of the maternal, paternal, and cord serum samples (90%–100%). PFNA, PFDA, PFUnDA, and PFDoDA (C9–C12) were detected in > 50% of the maternal serum samples, while detection frequencies of these compounds were < 3% in the cord serum samples. On the other hand, PFTrDA and PFTeDA (C13–C14) were rarely detected in maternal serum samples (2%–8%), while these compounds were detected in > 50% of the cord serum samples. The sum of PFAS concentrations (∑PFASs) was comparable between maternal and paternal serum (geometric means of 5.02 and 5.11 ng/mL, respectively), but ∑PFASs in cord serum showed a lower geometric mean (0.87 ng/mL) than those of maternal and paternal serum.

Within the same serum type, Spearman’s correlation was significant among the PFAS compounds (Supplemental Figure S1). Significant correlations were observed for several pairs of PFASs between maternal and cord serum, though none of the PFASs in maternal serum were significantly correlated with those in paternal serum. However, PFOA of cord serum showed positive correlation with PFOA, PFDA, PFUnDA, and PFDoDA in paternal serum (Supplemental Figure S1).

### 3.3. Composition Profiles of PFASs in Maternal, Paternal, and Cord Serum

**Figure 1:**
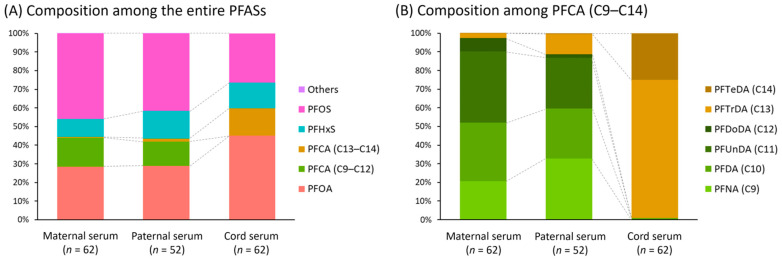
Composition of perfluoroalkyl substances (PFASs) in maternal, paternal, and cord serum. (**A**) Composition among the entire PFASs. (**B**) Composition among PFCA with carbon-chain lengths of 9 to 14 (C9–C12). “Others” in Figure 1A includes PFPeA, PFHxA, PFHpA, and PFBS.

PFOS was the dominant PFAS in maternal and paternal serum, which accounted for 46% and 41% of ∑PFASs, respectively (Figure 1). However, this dominance of PFOS diminished in cord serum with a contribution of 26%. The second dominant PFAS in maternal and paternal serum was PFOA (28% and 29%, respectively), but PFOA was the most dominant PFAS in cord serum with a 46% contribution. The contribution of PFCAs with carbon numbers from 9 to 12 ranged from 13% to 16% in maternal and paternal serum, but their contribution in cord serum was minuscule. On the other hand, PFCAs with longer carbon numbers (C13–C14) contributed to 15% of ∑PFASs, while their contribution in maternal and paternal serum was < 2%. The composition of PFASs in maternal and paternal serum was generally similar. However, the contribution of PFHxS was higher in paternal serum than in maternal serum (15% and 10%, respectively), while the contribution of PFCAs with carbon numbers from 9 to 12 was similar in maternal serum and paternal serum (16% and 13%, respectively).

### 3.4. Placental Transfer Rates and Their Determinants

**Figure 2:**
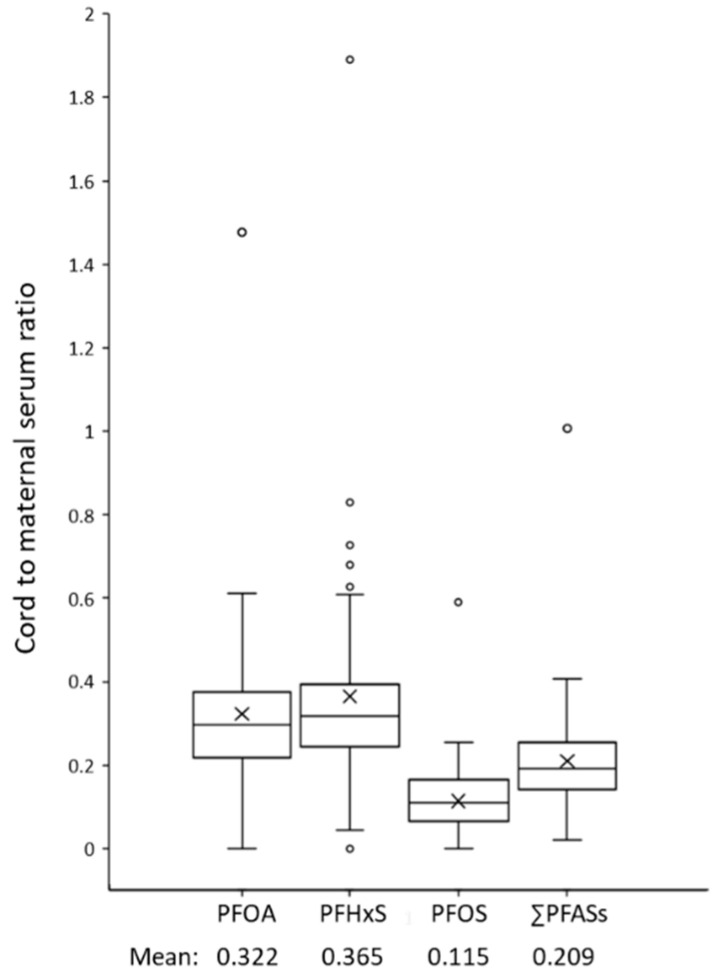
Box plots showing distribution of placental transfer rate (cord to maternal serum ratio) of perfluoroalkyl substances (PFASs) in the study population (*n* = 62). Median (line within box), first and third quartiles (bottom and top of box, respectively), 5th and 75th percentiles (lower and upper bars, respectively), and arithmetic mean (X) are shown.

Among PFOA, PFOS, and PFHxS, PFHxS showed the highest placental transfer rate (cord to maternal serum ratio) of 0.365. Placental transfer rates of PFOA and PFOS were 0.322 and 0.115, respectively (Figure 2). Placental transfer rates of other PFASs could not be calculated because of their low detection frequencies (e.g., < 5% of PFNA, PFDA, PFUnDA, and PFDoDA in cord serum; < 10% of PFTrDA and PFTeDA in maternal serum).

Although it was not possible to calculate placental transfer rates of several PFCAs due to their low detection frequencies in maternal or cord serum, our observations are in line with the U-shaped trend of the placental transfer rates with the increasing chain lengths of PFCAs. PFOA (C8) was detected in most of the maternal and cord serum with a placental transfer rate of 0.32, but PFNA, PFDA, PFUnDA, and PFDoDA (C9–C12) were detected in ≥ 60% of the maternal serum while these PFCAs were not detected in the cord serum, suggesting low placental transfer of the compounds (Table 2). On the other hand, the opposite trends in PFTrDA and PFTeDA (C13–C14), i.e., low detection frequencies in maternal serum (8% and 2%, respectively) and high detection frequencies in cord serum (55% and 52%, respectively), suggest relatively high placental transfer of the PFCAs with longer carbon chains.

When comparing placental transfer rates of PFOA, PFHxS, and PFOS, PFOA showed comparable yet slightly higher placental transfer rate than PFHxS in most studies, while the placental transfer rate of PFOA was slightly lower than that of PFHxS in our study (Table 3). In most of the studies including the present study, PFOS showed the lowest placental transfer rate among the three compounds.

Several variables related to pregnancy and birth outcome were associated with placental transfer rates of PFASs (Table 4). Maternal age was significantly associated with a decreased placental transfer rate of ∑PFASs. Maternal BMI before pregnancy was associated with a decreased placental transfer rate of PFOS, and maternal weight before pregnancy was also associated with those of PFOS and ∑PFASs. Birth weight and gestational age were positively associated with the placental transfer rate of PFOA, PFOS, and ∑PFASs.

## 4. Discussion

The PFAS concentrations in maternal, paternal, and cord serum measured in this study were smaller than those of previous Korean studies. A previous study on Korean pregnant women reported slightly higher concentrations in maternal or cord serum than those of the present study for several PFASs, but the PFAS concentrations of the two studies were mostly comparable [41]. On the other hand, in another study on Korean pregnant women, the concentrations of PFASs were much higher than those of the present study; median concentrations in maternal serum were 2.62, 1.21, and 9.37 ng/mL for PFOA, PFHxS, and PFOS, respectively, and median concentrations in cord serum were 2.08, 0.57, and 3.18 ng/mL for PFOA, PFHxS, and PFOS, respectively [47]. The concentrations of PFASs in paternal serum in the current study were lower than those in a previous study on the general population of Korea [50]. The reason why PFASs concentrations in maternal and cord serum were lower than those in previous studies is not clear, but the recent sampling of the sera in this study (2013–2015 in this study vs. 2008–2009 and 2011 in the previous studies) may be one reason for the reduced PFASs concentrations [14,15], taking into consideration the passage of time and decrease of the legacy PFASs or long-chain PFASs in the environment.

The trends of the PFAS composition in maternal and cord serum in this study are similar to those of previous studies. PFOA contribution was higher in maternal serum than in cord serum in the previous studies, while PFOS contribution was higher in cord serum than in maternal serum [49,51,52]. In addition, PFTrDA (C13) contribution was higher in cord serum than in maternal serum [49,51], which is in line with our observation.

There are a few studies comparing the concentration of PFAS between maternal, paternal, and cord serum. These include a Chinese cohort study in Shandong [53], two studies with pre-pregnant couples [30,54], and a selected population study [55]. Compared with the female partner, male partners had higher median concentrations for most of the PFAS and positive correlations were noted between the concentrations of the studied PFAS in plasma and in pre-pregnant couples [30,54]. In cohort study, the serum concentration of PFOA, PFOS, PFNA, PFUnDA, and PFHxS in the father had higher levels than in the mother [53]. Additionally, 10 PFASs were significantly correlated in matched maternal and cord serums, while most PFASs were correlated in paternal and maternal serums (r = 0.23–0.45) or paternal and cord serums from the same family (r = 0.14–0.45). Most PFASs, excluding PFBS and PFHpA, were significantly correlated in paired paternal and cord serums (r = 0.14–0.45) [53]. In our study, even though the detection frequencies of PFNA, PFDA, and PFUnDA in paternal serum were smaller than those in the maternal serum, the differences in the median were not big between the two serum types. Overall, concentrations of individual PFAS, sum of PFCA, sum of PFSA, and sum of PFAS were comparable between maternal and paternal serum, which is inconsistent with previous studies.

In this study, significant correlations were observed for several pairs of PFASs between maternal and cord serum, though none of the PFASs in maternal serum were significantly correlated with those in paternal serum. Considering that lifestyle is often shared by family members, exposure to PFASs within family is expected to have high correlations. The insignificant correlations between maternal and paternal PFAS concentrations observed in our study imply that the partners share common exposure sources to PFASs less. However, this explanation cannot explain the significant correlations of PFOA in cord serum with several PFASs in paternal serum. We cannot rule out a possibility of a statistical artifact due to the limited samples size (*n* = 52). Therefore, further studies with a larger number of paired samples should be investigated.

The composition of paternal PFASs was similar to that in maternal serum, but longer chain PFASs such as PFTrDA (C13) showed different contributions; PFTrDA was detected in 8% of maternal serum but detected in higher proportions of paternal and cord serum (65% and 55%, respectively). This higher proportion of PFTrDA in paternal and cord serum and the low proportion in maternal serum can be explained by placental transfer of this chemical. We can hypothesize that the concentration of PFTrDA in maternal serum before pregnancy was comparable to those in paternal serum, and PFTrDA in a high amount of the maternal serum was transferred to the fetuses during the pregnancy, which consequently led to decreased PFTrDA concentration in maternal serum compared to that in paternal and cord serum. Although there have been previous studies investigating the concentrations and correlations of PFASs within same family, to our knowledge, this study is the first to evaluate the composition of PFASs in maternal, paternal, and cord serum. Comparing composition of maternal, paternal, and cord serum provides significant epidemiologic data to evaluate exposure for different groups such as pregnant women, fetuses, and adult males.

Placental transfer rates of PFASs have been known to depend on their functional groups (e.g., carboxylic and sulfonic acids) and the length of the carbon chain [25,26,42]. For example, among the PFCAs, the placental transfer rates and the chain length showed U-shaped relationships in previous studies, and PFNA, PFDA, and PFUnDA (C9–C11) exhibited lower placental transfer rates than other PFACs [26,48]. The different binding affinity of PFASs to serum albumin explains the chemical structure-dependent placental transfer rates of PFASs. It is known that most PFASs in the blood are bound to serum protein, and albumin is the most important carrier of PFASs among the serum proteins [56]. Gao et al. hypothesized that PFASs with weaker binding affinity to serum albumin leads to their higher free fraction in blood, which leads to the concentration gradient of the free PFASs between maternal and fetal serum, and consequently facilitates the passive diffusion of PFASs through the placenta barrier [51]. This hypothesis was supported by the correlation between the dissociation constant of the serum albumin–PFAS complexes and the placental transfer rate of PFASs among Chinese pregnant women [51].

The associations of gestational age with placental transfer rates of PFASs are in line with observations of previous studies. In serum and placenta samples collected from women who had terminated their pregnancy before gestational week 12, the placenta-to-maternal serum ratios of PFOS, PFOA, and PFNA concentrations increased with increasing gestational age [57]. Similarly, fetus-to-maternal plasma ratios of PFOS, PFNA, and PFUnDA increased with increasing fetal age among the maternal and fetal samples collected from women who had terminated their pregnancy [32]. In a Chinese birth cohort study, placental transfer rates were higher in women with full-term delivery than women with preterm delivery for most PFASs [44]. Among the women with full-term delivery, these placental transfer rates were positively associated with expression levels of mRNAs encoding transporter proteins involving active transport of endogenous and exogenous substances across the placental barrier, while the association was not significant among the women with preterm delivery [44]. This different association with gene expression of the transporters may explain the increase in placental transfer rates with increasing gestational age. As with gestational age, an increase in birth weight shows an increase in placental transfer rates in our study. Low birth weight is the most consistently reported negative pregnancy outcome associated with prenatal exposure to PFASs in human epidemiologic and animal studies [23,58,59,60]. However, as the birth weight increased, the placental transfer rates of PFASs increased in our study. Although our study did not show a positive association between the placental weight and placental transfer rate of PFASs, the birth weight and placental weight both increased with increasing the gestational age [61], and the positive association between birth weight and placenta transfer rate in this study showed the same direction as gestational age. The relationship between gestational exposure to PFAS, placental function, and birth weight is the main topic for PFAS exposure on birth outcomes and should be further investigated.

The reason for the inverse associations of maternal age, pre-pregnant BMI, or pre-pregnant weight with placental transfer rates observed in this study is unclear. Pan et al. hypothesized that the difference in placental function by maternal age might be an explanation for the difference in placental transfer rate by maternal age, but the association between maternal age and placental transfer rate in this study had the opposite direction [40]. In previous studies, there was no association between pre-pregnant BMI and placental transfer rates of PFASs [39,40]. The inverse associations of pre-pregnancy BMI or weight with PFASs might be confounded by other factors such as maternal age or gestational age, but these associations were not statistically tested (e.g., multivariable regression) considering the limited sample size of our study population.

Based on the inverse association of maternal age with placental transfer rates observed in the present study, we hypothesize that placental changes with increasing maternal age might be related to placental transfer. There are many studies of placental changes with increasing maternal age like vascular malperfusion [62,63] and defects of decidualization and placentation [64]. The rate of maternal blood flow through the intervillous space and area available for exchange across the villous trophoblast epithelium are important variables for regulation of placenta transfer [65]. Advanced maternal age was associated with a higher rate of placental maternal vascular lesions [62,63], which is presumed to negatively affect maternal blood flow in the intervillous space and area available for exchange on villous trophoblast epithelium related to placenta transfer. Pan et al. also hypothesized that the difference in placental function by maternal age might be an explanation for the difference in placental transfer rate by maternal age, but the result was in the opposite direction [40]. There are few studies of association with placental transfer rate and maternal age, and the results have not shown consistent results. Further studies with more large-scale subject populations are needed.

This study has several limitations. First, the sample size of the study population was relatively small, which may limit statistical power and generalizability, although the results of this study are generally in line with previous studies. Second, our study attempted to compare compositions of PFAS in maternal, paternal, and cord serum, but 10 paternal serum were insufficient to analyze PFAS. Therefore, it is necessary to perform a larger study without missing data for clarification of the correlation between maternal, paternal, and cord serum. Third, inclusion of multiple variables in the regression model may cause an overfitting issue when the sample size is small. Therefore, it should be noted with caution that confounders could not be considered in the linear regression models considering the limited sample size. Forth, detection frequencies of several PFAS were relatively low compared with results of previous studies, which also limited interpretation of the results of this study. Low serum concentrations of PFASs as well as high LODs were two reasons why PFASs showed low detection frequencies; the LODs in the present study (0.03–0.08 ng/mL) were lower than the LODs or limit of quantification of several previous studies, showing a range of 0.003–0.038 ng/mL [26,49,51]. Fifth, PFOA, PFOS, and PFHxS exist in their different isomer forms (i.e., linear and branched forms) in human serum [43,45], but PFASs isomers were not separated in our chemical analysis method. Since the isomers may pose different placental transfer properties due to their difference in chemical structure, future analysis with isomer separation would provide more information. Sixth, although the title shows PFAS, we did not measure all compounds of PFCA and PFSA; only 13 perfluoroalkyl acids were analyzed. Seventh, exposure to novel PFASs has recently become an issue of public health interest [26,46], but we could not measure these novel chemicals.

## 5. Conclusions

The results of the present study showed different composition profiles of PFASs among maternal, paternal, and cord serum. To our knowledge, this study is the first to evaluate the composition of PFASs in maternal, paternal, and cord serum.

Placental transfer rates of PFASs differ by compounds, and several factors such as gestational age were identified to be associated with the placental transfer of PFASs. Recent developments in chemical analysis have discovered novel PFASs and separately measured PFAS isomers in human samples. It is warranted to investigate concentrations of these novel chemicals in maternal and cord serum and their placental transfer rates, which has not yet been studied for Korean pregnant women.

## Figures and Tables

**Table 1:** Characteristics of the study population.

	*n* (%)	Arithmetic Mean ± SD (Range) ^a^
Maternal Variables		
Maternal age (year)	62 (100)	32 ± 3 (26–41)
Maternal education		
≤12 year	11 (18)	
>12 year	51 (82)	
Family income		
<3000 USD/month	30 (48)	
≥3000 USD/month	32 (52)	
Smoking history		
Never smoker	58 (94)	
Former smoker	4 (6)	
Current smoker	0 (0)	
Pre-pregnant BMI (kg/m^2^) ^b^	62 (100)	22.0 ± 3.9 (16.6–32.2)
Pre-pregnant weight (kg)	62 (100)	57.4 ± 11.0 (43.0–95.0)
Parity		
0	34 (55)	
1	21 (34)	
≥2	7 (11)	
Paternal Variable ^c^		
Paternal age (year)	52 (100)	34 ± 4 (27–41)
Paternal education		
≤12 year	11 (21)	
>12 year	41 (79)	
Smoking history		
Never smoker	14 (27)	
Former smoker	21 (40)	
Current smoker	17 (33)	
Birth Outcome		
Birth weight (kg)	62 (100)	2.94 ± 0.56 (1.71–3.94)
Placental weight (g)	58 (94) ^d^	567 ± 127 (335–800)
Gestational age (week)	62 (100)	37.5 ± 2.5 (30.6–41.0)
Infant sex		
Male	35 (56)	
Female	27 (44)	

^a^ Only arithmetic mean ± standard deviation (SD) and range (minimum–maximum) of continuous variables were shown. ^b^ Body mass index = weight (kg)/[height (m)]^2^. ^c^ 10 participants with missing values for paternal variables. ^d^ Placental weight has four missing observations.

**Table 2:** Detection frequencies and concentrations (ng/mL) of perfluoroalkyl substances (PFASs) in maternal, paternal, and cord serum.

Compound	Serum Type	*n*	Detection Frequency (%)	GM ± SD ^a^	Percentiles		
					25th	50th	75th
PFOA	Maternal serum	62	100	1.41 ± 1.63	1.08	1.32	1.96
	Paternal serum	52	100	1.44 ± 1.92	0.98	1.29	1.79
	Cord serum	62	98	0.40 ± 2.03	0.30	0.41	0.61
PFNA	Maternal serum	62	85	0.15 ± 2.67	0.04	0.14	0.25
	Paternal serum	52	56	-	<0.03	0.13	0.37
	Cord serum	62	0	-	<0.03	<0.03	<0.03
PFDA	Maternal serum	62	100	0.26 ± 1.58	0.19	0.26	0.33
	Paternal serum	52	90	0.21 ± 2.00	0.11	0.18	0.28
	Cord serum	62	3	-	<0.06	<0.06	<0.06
PFUnDA	Maternal serum	62	92	0.32 ± 1.85	0.17	0.29	0.52
	Paternal serum	52	69	-	<0.05	0.16	0.28
	Cord serum	62	0	-	<0.05	<0.05	<0.05
PFDoDA	Maternal serum	62	60	-	<0.07	0.08	0.10
	Paternal serum	52	10	-	<0.07	<0.07	<0.07
	Cord serum	62	0	-	<0.07	<0.07	<0.07
PFTrDA	Maternal serum	62	8	-	<0.05	<0.05	<0.05
	Paternal serum	52	65	-	<0.05	0.06	0.15
	Cord serum	62	55	-	<0.05	0.14	0.22
PFTeDA	Maternal serum	62	2	-	<0.03	<0.03	<0.03
	Paternal serum	52	10	-	<0.03	<0.03	<0.03
	Cord serum	62	52	-	<0.03	0.03	0.06
PFHxS	Maternal serum	62	100	-	0.32	0.44	0.60
	Paternal serum	52	100	-	0.61	0.72	0.87
	Cord serum	62	97	-	0.10	0.15	0.20
PFOS	Maternal serum	62	100	2.31 ± 1.58	1.75	2.35	3.18
	Paternal serum	52	100	2.21 ± 1.84	1.48	2.04	3.05
	Cord serum	62	90	0.25 ± 2.03	0.13	0.26	0.39
∑PFCAs ^b^	Maternal serum	62	-	2.17 ± 1.72	1.62	2.07	2.95
	Paternal serum	52	-	2.01 ± 2.12	1.27	1.75	2.92
	Cord serum	62	-	0.51 ± 2.24	0.37	0.62	0.89
∑PFSAs ^c^	Maternal serum	62	-	2.79 ± 1.57	2.01	2.87	3.63
	Paternal serum	52	-	3.05 ± 1.78	2.12	2.78	3.82
	Cord serum	62	-	0.36 ± 2.07	0.25	0.42	0.61
∑PFASs ^d^	Maternal serum	62	-	5.02 ± 1.61	3.76	5.19	6.30
	Paternal serum	52	-	5.11 ± 1.90	3.51	4.59	6.47
	Cord serum	62	-	0.87 ± 2.15	0.65	1.02	1.55

^a^ Geometric mean (GM) and standard deviation (SD) of PFAS with detection frequency < 70% were not calculated. Detection frequencies of PFPeA, PFHxA, PFHpA, and PFBS were < 2% in all serum types. ^b^ Sum of concentrations of PFPeA, PFHxA, PFHpA, PFOA, PFNA, PFDA, PFUnDA, PFDoDA, PFTrDA, and PFTeDA. ^c^ Sum of concentrations of PFBS, PFHxS, and PFOS. ^d^ Sum of concentrations all PFASs.

**Table 3:** Placental transfer rates of PFASs in the previous literature and this study.

Reference	Country	Sample Size	Blood Type	Sampling of Maternal Blood	Placental Transfer Rate ^b^		
					PFOA	PFHxS	PFOS
Wang et al., 2019 [21]	China	369	Serum	Not specified	0.83	**0.95**	0.30
Manzano-Salgado et al., 2015 [39]	Spain	53–66	Serum	12nd week	**1.90**	0.40	1.86
Pan et al., 2017 [40]	China	≤100	Serum	1st, 2nd, or 3rd trimester	**0.66**	0.49	0.35
Kim et al., 2011 [41] ^a^	Korea	≤44	Serum	Mostly during the third trimester of pregnancy, but several subjects were sampled earlier	**1.02**	0.72	0.48
Eryasa et al., 2019 [42]	FaroeIslands	151	Serum	32nd week	**0.80**	0.62	0.38
Zhao et al., 2017 [43]	China	≤63	Blood	3rd trimester or within the 1st week after delivery	**0.57**	0.35	0.20
Cai et al., 2020 [26]	China	≤423	Serum	Within 1 week before delivery	**0.73**	0.46	0.42
Li, Cai et al., 2020 [44]	China	≤187	Serum	Within 1 week before delivery	**0.85**	0.72	0.58
Chen et al., 2017 [45]	China	≤32	Serum	Within 3 days before delivery	**0.77**	0.55	0.40
Li, Yu, et al., 2020 [46]	China	117	Serum	Within 3 days before delivery	**1.03**	0.83	0.46
Lee et al., 2013 [47]	Korea	59	Serum	At delivery	**0.80**	0.50	0.32
Zhang et al., 2013 [48]	China	30	Blood	Within 1 hour of delivery	**0.57**	0.32	0.18
Liu et al., 2011 [49]	China	≤50	Serum	Within the first week after delivery	**0.89**	0.73	0.54
This study	Korea	62	Serum	At delivery	0.32	**0.36**	0.12

^a^ Mean values were presented. ^b^ Boldface represents the highest placental transfer rate among those of PFOA, PFHxS, and PFOS, whereas underline represents the lowest placental transfer rate.

**Table 4:** Difference (95% CI) in placental transfer rate (cord to maternal serum ratio) of perfluoroalkyl substances (PFASs) associated with potential determinants in the study population (*n* = 62) ^a^.

Potential Determinant	PFOA	PFHxS	PFOS	∑PFCAs	∑PFSAs	∑PFASs
Maternal age (IQR: 30–34 y) ^b^	−0.05 (−0.11, 0.01)	−0.04 (−0.11, 0.04)	−0.03 (−0.05, 0.00)	−0.06 (−0.11, −0.01) *	−0.03 (−0.06, 0.00)	−0.04 (−0.08, 0.00) *
Pre-pregnant BMI (IQR: 19.7–23.9 kg/m^2^) ^b^	−0.05 (−0.10, 0.01)	−0.05 (−0.12, 0.02)	−0.03 (−0.05, 0.00) *	−0.05 (−0.09, 0.00) *	−0.03 (−0.05, 0.00)	−0.04 (−0.07, 0.00)
Pre-pregnant weight (IQR: 50–62 kg) ^b^	−0.05 (−0.11, 0.00)	−0.06 (−0.13, 0.01)	−0.03 (−0.05, 0.00) *	−0.06 (−0.10, 0.00) *	−0.03 (−0.06, 0.00) *	−0.04 (−0.08, 0.00) *
Weight gain (IQR: 8–17 kg) ^b^	0.05 (−0.03, 0.14)	−0.02 (−0.08, 0.13)	0.02 (−0.02, 0.06)	0.05 (−0.02, 0.12)	0.02 (−0.02, 0.06)	0.03 (−0.02, 0.09)
Birth weight (IQR: 2.56–3.38 kg) ^b^	0.10 (0.04, 0.18) **	0.09 (−0.01, 0.18)	0.04 (0.01, 0.08) **	0.07 (0.01, 0.13) *	0.05 (0.01, 0.08) *	0.06 (0.01, 0.11) *
Placental weight (IQR: 478–650 g) ^b^	0.04 (−0.03, 0.12)	0.07 (−0.03, 0.16)	0.02 (−0.01, 0.06)	0.03 (−0.03, 0.10)	0.03 (−0.01, 0.06)	0.03 (−0.02, 0.08)
Gestational age (IQR: 35.4–39.6 wk) ^b^	0.12 (0.03, 0.20) **	0.10 (−0.01, 0.20)	0.04 (0.01, 0.08) *	0.07 (0.00, 0.14)	0.05 (0.00, 0.09) *	0.06 (0.00, 0.12) *
Infant sex (female vs. male) ^c^	0.01 (−0.10, 0.11)	−0.03 (−0.16, 0.10)	0.00 (−0.05, 0.04)	0.02 (−0.07, 0.11)	0.00 (−0.06, 0.05)	0.01 (−0.06, 0.08)
Parity (≥ 1 vs. 0) ^c^	−0.07 (−0.18, 0.03)	−0.01 (−0.14, 0.12)	−0.02 (−0.07, 0.02)	−0.05 (−0.14, 0.04)	−0.03 (−0.08, 0.03)	−0.04 (−0.11, 0.02)

^a^*n* = 58 for placental weight. ^b^ A difference in placental transfer rate of PFAS by an interquartile range (IQR) increase in each variable was represented. ^c^ A difference in placental transfer rate of PFAS between the groups was represented. * *p* < 0.05; ** *p* < 0.01.

## Data Availability

The datasets generated and analyzed during the current study are not publicly available due to protection of patient privacy but are available from the corresponding author on reasonable request.

## Supplementary Materials

The following are available online at https://www.mdpi.com/article/10.3390/toxics9070168/s1, Table S1: MS/MS parameters for determination of perfluoroalkyl substances (PFASs) in serum; Table S2: LC-MS/MS conditions for determination of perfluoroalkyl substances (PFASs) in serum; Table S3: Limits of detection (LODs) of perfluoroalkyl substances (PFASs). Figure S1: Spearman’s correlation among perfluoroalkyl substances (PFASs) in maternal, paternal, and cord serum.
