# Supplementary material for: Placental Transfer and Composition of Perfluoroalkyl Substances (PFASs): A Korean Birth Panel of Parent-Infant Triads
Source: Toxics. 2021 Jul 14;9(7):168. doi: 10.3390/toxics9070168 (PMC8309930; doi:10.3390/toxics9070168)
Supplement: Supplementary file 1 [file toxics-09-00168-s001.zip › toxics-1253777 - supplemmentary - final.pdf]

toxics

Article

Supplementary Material: Placental Transfer and Composition of Perfluoroalkyl Substances (PFASs): A Korean Birth Panel of Parent-Infant Triads

Habyeong Kang, Hee-Sun Kim, Yeong Sook Yoon, Jeongsun Lee, Younglim Kho, Jisun Lee, Hye Jin Chang, Yoon Hee Cho and Young Ah Kim

Supplementary Materials and Methods

Table S1. MS/MS parameters for determination of perfluoroalkyl substances (PFASs) in serum.

Compound	Precursor Ion (m/z)	Product Ions (m/z)	Collision Energy (V)
PFPeA	263.0	218.9	−12
PFHxA	313.0	268.9<br>119.0	−12<br>−30
PFHpA	363.0	318.9<br>168.9	−14<br>−24
PFOA	413.0	368.9<br>168.8	−16<br>−26
PFNA	463.0	418.8<br>218.9	−14<br>−26
PFDA	513.0	468.9<br>218.9	−16<br>−26
PFUnDA	562.9	518.9<br>268.8	−18<br>−26
PFDoDA	613.0	569.0<br>168.8	−18<br>−40
PFTrDA	663.0	619.1<br>168.9	−16<br>−44
PFTeDA	713.1	669.1<br>168.9	−20<br>−44
PFBS	298.9	79.8<br>99.0	−60<br>−42
PFHxS	399.0	79.9<br>98.9	−78<br>−54
PFOS	499.0	80.1<br>99.0	−92<br>−70

Abbreviations: tandem mass spectrometry, MS/MS.

Table S2. LC-MS/MS conditions for determination of perfluoroalkyl substances (PFASs) in serum.

Parameter		Condition					
HPLC	Column	YMC-Pack ODS-AQ, 2.0 × 150 mm, 3.0 µm					
	Mobile phase	A: 5 mM ammonium acetate and 0.02% formic acid in water<br>B: methanol					
	Gradient	Time (min)	0	5	13	13.1	25
		A (%)	70	0	0	70	70
		B (%)	30	100	100	30	30
	Flow rate	200 µL/min					
	Injection volume	3 µL					
MS/MS	Mode	ESI negative					
	Curtain Gas	25 psi					
	Gas temperature	400 °C					
	Ion spray voltage	−4000 kV					
	Ion source gas 1	40 psi					
	Ion source gas 2	60 psi					
	Collision gas (CAD)	6					

Abbreviations: high-performance lipid chromatography, HPLC; tandem mass spectrometry, MS/MS.

Table S3. Limits of detection (LODs) of perfluoroalkyl substances (PFASs).

Compound	LOD (ng/mL)
PFPeA	0.07
PFHxA	0.06
PFHpA	0.05
PFOA	0.05
PFNA	0.03
PFDA	0.06
PFUnDA	0.05
PFDoDA	0.07
PFTrDA	0.05
PFTeDA	0.03
PFBS	0.08
PFHxS	0.05
PFOS	0.03

Supplementary Results

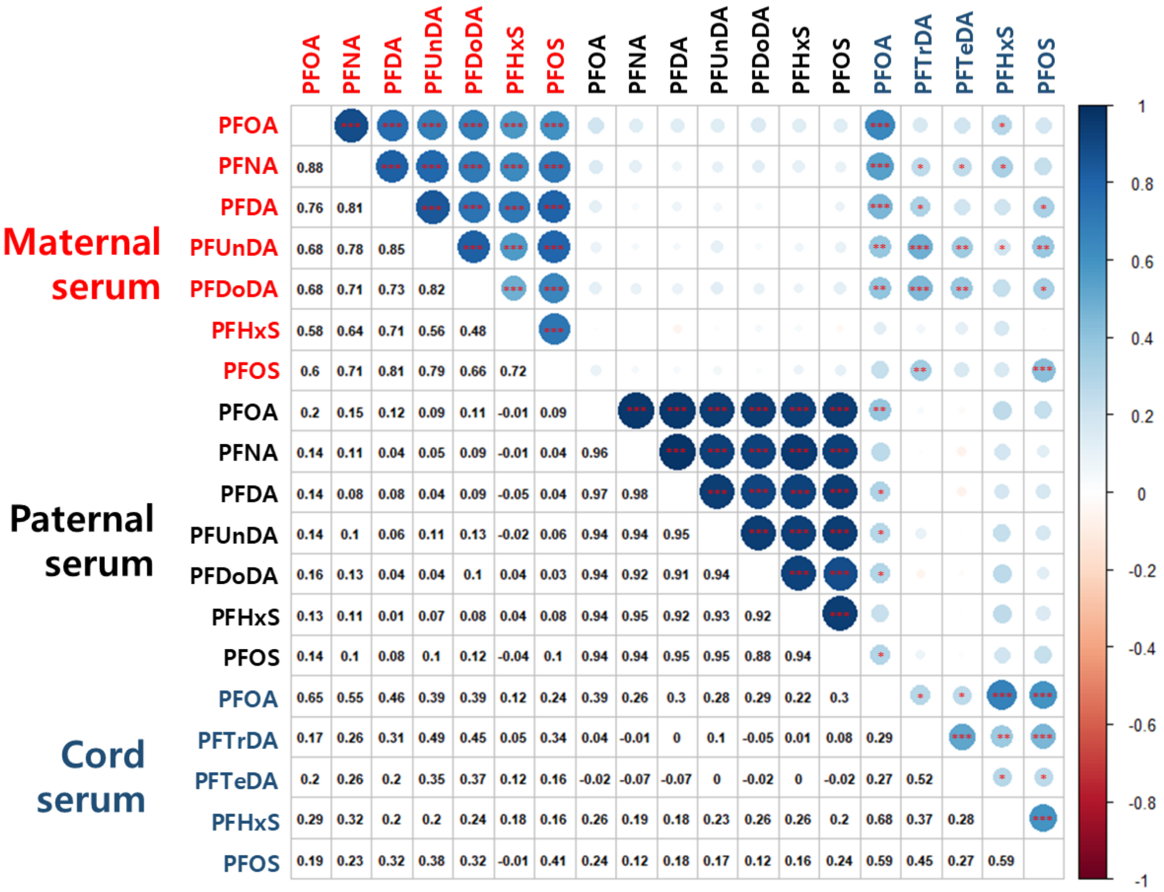

Figure S1. Spearman's correlation among perfluoroalkyl substances (PFASs) in maternal, paternal, and cord serum. The size and the color of the circles represent the correlation coefficients. Asterisks (*, **, and ***) on the circles represent $p < 0.05$, $p < 0.01$, and $p < 0.001$, respectively.
